# What determines eukaryotic translation elongation: recent molecular and quantitative analyses of protein synthesis

**DOI:** 10.1098/rsob.200292

**Published:** 2020-12-09

**Authors:** Nagammal Neelagandan, Irene Lamberti, Hugo J. F. Carvalho, Cédric Gobet, Felix Naef

**Affiliations:** Institute of Bioengineering, School of Life Sciences, Ecole Polytechnique Fédérale de Lausanne (EPFL), Lausanne CH-1015, Switzerland

**Keywords:** protein synthesis, translation elongation, eIF5A, mathematical modelling, TASEP, SunTag imaging

## Abstract

Protein synthesis from mRNA is an energy-intensive and tightly controlled cellular process. Translation elongation is a well-coordinated, multifactorial step in translation that undergoes dynamic regulation owing to cellular state and environmental determinants. Recent studies involving genome-wide approaches have uncovered some crucial aspects of translation elongation including the mRNA itself and the nascent polypeptide chain. Additionally, these studies have fuelled quantitative and mathematical modelling of translation elongation. In this review, we provide a comprehensive overview of the key determinants of translation elongation. We discuss consequences of ribosome stalling or collision, and how the cells regulate translation in case of such events. Next, we review theoretical approaches and widely used mathematical models that have become an essential ingredient to interpret complex molecular datasets and study translation dynamics quantitatively. Finally, we review recent advances in live-cell reporter and related analysis techniques, to monitor the translation dynamics of single cells and single-mRNA molecules in real time.

## 1. Introduction

Translation of genetic information into functional proteins is a fundamental process across all forms of life, representing the critical step of gene expression. It allows cells and tissues to maintain homeostasis and to react to external signals, and its dysregulation underlies multiple diseases. Even though the process has been known since the 1950s, its experimental and analytical studies have presented major challenges until today. Lately, the advent of new approaches, notably ribosome profiling (ribo-seq), have provided the unprecedented opportunity to monitor the process of translation *in vivo*, providing genome-wide and codon-resolved insights into the different steps of translation, as well as identification of their main determinants and regulatory mechanisms in several organisms. Specifically, in this review, we aim to provide an overview of the latest studies on the mechanism of translation elongation in mammals. We will focus on mature mRNA being actively translated and not on pioneering rounds of translation, which can substantially differ from other later rounds and have been covered in other reviews [1]. The research works presented here contribute to answering some important questions related to translation elongation: what determines the net production rate of a specific protein? How is this rate regulated by the cell? In which ways does the codon sequence influence or determine initiation and elongation rates and, consequently, protein synthesis?

Protein synthesis occurs in four major steps: initiation, elongation, termination and ribosome recycling. In translation initiation, various proteins called initiation factors (eIFs) facilitate proper assembly of 40S and 60S ribosomal subunits to form the 80S complex at the mRNA start codon with an initiator methionyl-tRNA bound to the P-site [2]. In the next step of translation elongation, the 80S complex moves along the mRNA, three nucleotides at a time, extending the encoded protein, in coordination with various elongation factors (eEFs) and aminoacyl-tRNAs (aa-tRNAs). When the 80S complex reaches the termination codon, proteins called release factors (eRFs) are employed to facilitate the release of the nascent peptide. Finally, the post-termination ribosomes are split into 40S and 60S subunits to start a new round of translation.

Translation elongation is a complex process, requiring coordinated functioning of many components like the mRNA template, tRNAs, ribosomes, and many trans-acting factors and regulators. Earlier methods to study translation involved measuring protein output or amino acid incorporation, for example, using pulse-chase approaches. Polysome profiling was used as a ‘gold-standard’ to assess the global state of translating ribosomes on transcripts, as this separates mRNA based on the number of associated ribosomes on a sucrose density gradient [3]. More recently, various reporter assays uncovered certain key factors involved in translation, including the dynamic nature of the process, but such approaches are limited to the analysis of specific targets, typically using transgenes [4–6]. Ribo-seq, despite its inherent snapshot and bulk averaging, has proven itself to be a powerful tool to obtain genome-wide translation analyses, by measuring positions of translating ribosomes at nucleotide resolution. Its introduction has paved the way for a wealth of experiments, models and analyses in multiple organisms aiming at identifying the determinants of translational regulation [7]. In particular, it has revealed mRNA features like codon usage, codon context, secondary structures and amino acid sequences as modulators of translation elongation rates [8,9].

In combination with theoretical modelling and simulations, ribo-seq data have shown that initiation is the major rate-limiting step of translation in yeast, under normal conditions [10,11]. Under cellular stress or disease, global translation is repressed by shutting down cap-dependent initiation, while selective translation is achieved by cap-independent initiation [12]. In addition to the initiation, recent paradigm-shifting studies using ribo-seq in combination with other approaches [13] have emphasized the importance of translation elongation in protein synthesis. But ribo-seq cannot provide information on the heterogeneity at the single-molecule level. Moreover, being static (single time point measurement), it can only partially and/or indirectly uncover dynamical aspects of the process, except when it is combined with inhibitors like harringtonine and pulse-chase experiments.

Post-transcriptional modifications like mRNA methylation are known to modulate translation. m6A associated proteins YTHDF1 and METTL3 enhance translation efficiency by recruiting translation initiation factors to mRNA [14,15]. Studies using prokaryotic systems showed that this modification also affects translation elongation dynamics. mRNA secondary structure has also been shown to influence translation output. It was observed that the inclusion of modified nucleotides caused mRNA structural changes which in turn lead to changes in protein expression [16]. It was shown that 5′-leader sequences with reduced secondary structures undergo efficient translation and reporter assays showed an increased protein production from CDS with secondary structures. However, the effect of mRNA secondary structure on translation is still poorly understood and requires more extensive studies.

In this review, we will first discuss the main determinants of translation elongation rates (figure 2*a*) and how changes in these factors during stress and disease trigger specific cellular responses. Following this, we will review features leading to ribosome collisions or stalling and evolutionary conserved mechanisms to facilitate elongation and prevent (factors such as eIF5A) or clear these events (ribosome quality control, RQC). We will present recent advances in mathematical and computational models of translation and their importance for the analysis of ribo-seq and single-molecule translation reporter data. In particular, we will focus on a widely used class of models to study the dynamical properties of translation known as the totally asymmetric exclusion process (TASEP), and its variants. At present, a great majority of these theoretical and computational approaches have been applied in yeast, but these are likely to reveal novel insights into translation dynamics in higher eukaryotes including humans in the near future. Finally, we will describe recent advances in single-molecule dynamical measurements of translation, based on protein tagging and fluorescence imaging.

## 2. Major determinants of translation elongation

### 2.1. Influence of (aminoacyl-)tRNA abundance on translation elongation rates

**Figure 1.**
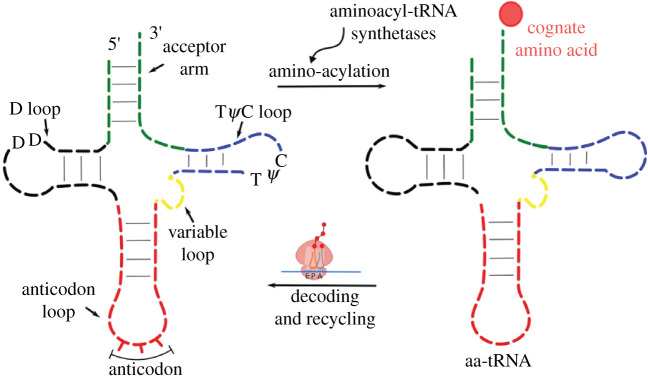
Structure and aminoacylation of tRNAs. Secondary structure of tRNA showing the different domains and the hairpin loops: the D-loop, with a variable content of dihydrouridine; the anticodon loop, containing the anticodon triplet; a variable region arranged in a stem loop; the T*ψ*C-loop with a sequence of thymine-pseudouridine-cytosine; the acceptor arm, 3'-end at which the amino acid is conjugated/bound. D, dihydrouridine; T, thymidine; *ψ*, pseudouridine; C, cytidine. tRNAs undergo aminoacylation with the help of enzymes called aminoacyl-tRNA synthetases; during translation tRNAs is de-aminoacylated, and then re-enters the pool of free tRNA to undergo another aminoacylation cycle.

Transfer RNAs (tRNAs) are key molecules of the cells' translational machinery, which allow decoding of codons into amino acids. They have a unique cloverleaf secondary structure with three hairpin loops. One of these loops, called the anticodon loop, recognizes the codons. The D-loop, close to the 5′-end of tRNA has a dihydrouridine base and the T*Ψ*C-loop close to the 3′-end has a sequence of thymine-pseudouridine-cytosine bases. The acceptor arm, at the 3′ of the tRNA, is where the amino acid is attached by enzymes called aminoacyl-tRNA synthetase (aaRS) [17] (figure 1). Indeed, the rate, efficiency and accuracy of translation are heavily influenced by the availability of aminoacyl-tRNAs (aa-tRNAs). Quantifying the levels of tRNAs is notoriously difficult due to the presence of many post-transcriptional modifications and secondary structures. Despite this, many efforts were made to create a comprehensive view of tRNA pools. Notably, the advent of high throughput methods to study tRNA levels has provided a greater understanding of tRNA landscapes in cells under different conditions [18–22].

A microarray-based approach has shown that tRNA pools differ among tissues in humans [23] and that there is a significant correlation between relative tRNA abundance and codon usage of highly expressed, tissue-specific genes [23]. Differences in tRNA pool composition between proliferating and differentiating cells were observed [24], which corresponds to distinct codon usage patterns. This study revealed that such coordination between tRNA and codon usage could be due to histone modifications around the tRNA genes. The existence of distinct tRNA pools during different cell cycle stages has been recently confirmed *in vitro*, alongside differences in codon usage, in particular in cells in G1 and G2/M phases [25]. Additionally, this study also revealed that during proliferation, translation of mRNAs enriched in rare codons is increased, and that this increase in decoding rate of rare codons could be attributed to the increase in the availability of ready-to-translate tRNAs, i.e. aa-tRNAs [25]. A recent report from our laboratory also indicates that there is a significant correlation between codon usage and tRNA availability in mouse liver tissues [18]. Taken together, emerging evidence highlights that the tRNA landscape varies between cellular states, which in turn leads to gene expression specificity by altering translation elongation parameters.

### 2.2. tRNA modifications

tRNAs are unique molecules known for their abundant modifications [26]. These modifications are known to influence their function and structure [27]. For example, the implications of these modifications and their complex nature were emphasized, when yeast and human cells m1A58 tRNA modifications exhibited different functions. It was shown to influence the stability and maturation of initiator-tRNA^met^ in yeast, while in human cells, this modification altered the tRNA association with polysome fractions in addition to affecting the stability [28,29]. Pseudouridylation, *ψ* (position 8), mediated by PUS7, influences general translation initiation [30]. In fact, tRNA fragments derived from the 5′-end of *ψ* containing tRNAs inhibit the translation initiation complex, which in turn can regulate early embryogenesis [30].

Several modifications have also been identified in the anticodon loop, which are known to affect the decoding efficiency of tRNAs. A genome-wide study using ribosome profiling in yeast and *C. elegans* has shown that loss of U34 modifications in the anticodon wobble position leads to ribosome pausing events and that the loss of such modifications leads to protein homeostasis defects which are rescued upon overexpression of hypomodified tRNA [31]. Modifications in and around the anticodon loop influence the interaction with cognate and near-cognate codons. Another modification, m5C38 mediated by DNMT2, has been shown to influence the accuracy of translation and this modification also protects tRNAs from ribonuclease activity [32]. Interestingly, it was shown that this m5C38 modification is dependent on queuosine (Q) modification occurring in the wobble anticodon position of tRNAs for amino acids His, Asn, Tyr and Asp. These two modifications together regulate the translation rate of the cognate and near-cognate Q-decoded codons and protect the tRNAs from ribonucleolytic cleavage [33].

Modifications in the wobble position (position 34) and the immediate 3′-position of the anticodon (position 37) influence the decoding function of tRNAs affecting the accuracy and fidelity of translation [34]. Inosine at the wobble position (I34) is the result of deamination of adenosine by adenosine deaminase acting on transfer RNA (ADAT) enzyme. This I34 modification permits recognition of C, U and A nucleotides at the third position of codons thus leading to extended base-pairing capacity via non-Watson–Crick base-pairing [35]. A recent study in yeast showed that tRNA modification in the anticodon loop also plays a role in stop codon readthrough. In fact, loss of *ψ*35 and i6A37 modifications of Tyr tRNA caused a reduction in readthrough efficiency without affecting the decoding of tyrosine codons [36].

### 2.3. Codon usage and codon pairs

Synonymous codons are used at different frequencies, a property known as ‘codon usage bias’. Codon content of a translatome is one of the factors contributing to the large variability and codon-dependent heterogeneity in ribosome elongation rates. Indeed, indirect estimates of ribosome dwell times obtained from yeast and mouse liver ribosome profiling datasets have shown that ribosome dwell times, that is the time spent by a ribosome on a specific position on a transcript, are strongly codon-specific [18,37,38]. The suggestion that frequently used codons are translated faster is frequent in the literature, in many organisms. It has been proposed that codon usage bias could be either due to ‘mutation’ causing variation between species or due to ‘natural selection’ to improve translation efficiency across a genome, reviewed in [39]. The advent of ribosome profiling has uncovered various interesting aspects of codon usage and translation. Using ribosome profiling data from yeast, it was shown that synonymous codons have different decoding rates depending on their usage [40]. Additionally, cell-free translation assays and ribosome profiling in *Neurospora* showed that codon usage impacts elongation rates and that there is a negative correlation between ribosome occupancy and codon usage [41]. While there is more evidence of a relation between codon usage and ribosome occupancy in yeast, a similar relationship does not seem to be found in higher eukaryotes [42]. This was also reiterated using ribosome dwell time analysis on ribosome profiling data [18]. There was a strong correlation between dwell time and codon usage in yeast cells and this did not seem to be the case in mice. However, a previous report showed that this disconnect could be explained by GC mutational biases across genes [43].

When a ribosome is translating an mRNA, it interacts with more than one codon at a time. The effect of adjacent codon on translation elongation in eukaryotes was first shown in yeast [44]. This study identified 17 pairs of inhibitory codons that slowed elongation rates. The majority of these inhibitory codons involve wobble base-pairing leading to poor elongation. Dwell times could be influenced by codon pairs in mouse liver tissue and codon pair dwell times were found to be stable across the feeding/fasting cycle [18]. Another factor that influences translation elongation rates is amino acid composition of the peptide being synthesized. In fact, a recent study in yeast has shown that, because of interactions with the ribosome exit-tunnel, negatively charged amino acids are translated faster than positively charged ones and amino acids with smaller side chains are faster than the ones with bigger side chains [11].

### 2.4. Indirect influence of codon usage on mRNA stability

Various factors have been associated with mRNA stability and turnover (reviewed in [45,46]). Here, we highlight the role of codon usage—and thereby translation—in regulating the mRNA stability. Early evidence of a relationship between codon usage and mRNA stability was shown in yeast when a stretch of arginine codons enhanced polysome-associated mRNA decay [47], indicating a link between translation dynamics and mRNA stability. It was later shown that stable transcripts are more associated with optimal codons and substitution of these codons to non-optimal ones results in decreased mRNA stability [48].

Optimal codons are codons corresponding to the cognate tRNA species that are more abundant and undergo faster and efficient translation. It was also shown that codon optimality influences ribosomal translocation rate and thereby translation efficiency [48]. Further studies that followed up showed that this relationship between codon usage and RNA stability is preserved in higher organisms [49–53]. This relationship between codon usage of a transcript and mRNA stability is defined by the measure of codon stabilization coefficient (CSC). CSC is defined as the correlation coefficient between the frequency of occurrence of each codon in mRNA and the mRNA half-life [48]. CSC values have been found to correlate well with tRNA adaptation index (tAI), which is the metric showing how efficiently a codon is translated in the given tRNA pool [54].

A comprehensive study on CSCs in yeast showed that a big proportion of optimal codons was found for genes coding highly abundant proteins. In addition to that, a positive correlation was seen between protein abundance obtained from proteomics studies and CSC values [55]. Work using human cell lines indicates that the codons associated with mRNA instability have significantly longer dwell times in the A-site than in the P- and E-sites [52]. Strikingly, this interplay between translation elongation and mRNA stability could be regulated partially by intracellular tRNA and amino acid levels [49].

### 2.5. Deregulation of translation elongation during stress and disease

Cells are known to regulate the tRNA landscape and codon usage [21,24], as their imbalance can affect translation efficiency and elongation speed [56], compromising cellular homeostasis. Importantly, codon optimality was shown to play a key role in cellular stress. For example, during amino acid starvation in HEK293T cells, selective mRNA translation was achieved due to alteration in global codon usage: translation efficiency of mRNAs enriched in rare codons was increased alongside an enrichment in rare tRNA isoacceptors [57]. Codon optimality and tRNA levels are also heavily implicated in tumours. For example, a recent study taking advantage of the comprehensive TCGA database showed an altered tRNA landscape in different cancerous tissues, mainly driven by cellular proliferation state [58], which is similar to earlier observations [24]. Finally, mis-regulation of specific codons or amino acids has been also associated with cancer [58,59].

### 2.6. Codon features as determinants of ribosome stalling

As discussed above, dwell times or elongation rates of a translating ribosome vary along the mRNA. Depending on parameters such as codon optimality, peptide-bond formation efficiency, availability of elongation factors or nascent chain properties, ribosomes can have different dwell times. Sometimes the dwell time is so prolonged that the ribosome stops during elongation, a phenomenon referred to as *ribosome stalling*.

While in some cases ribosome stalling can be regulatory, as it has been described to promote correct protein translation, folding, complex assembly and targeting [60–62], recent studies are starting to uncover the fates of ‘non-intentionally' stalled ribosomes as signals that alert for the presence of defective mRNAs or altered physiological states. Although the causes and the fates of stalled ribosomes are not yet clear, the emergence of techniques that allow to map ribosomal stop sites [63,64] started to illustrate a complex picture, showing enrichment of certain motifs and revealing some factors that seem to be involved in the regulation of stalling.

**Figure 2.**
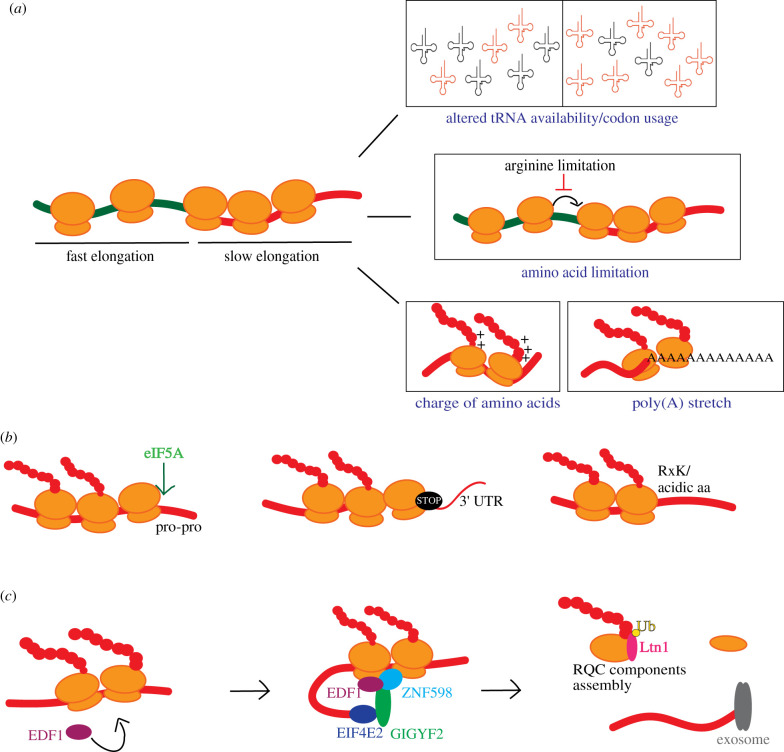
Factors influencing translation elongation and mRNA features causing ribosome stalling and rescue processes. (*a*) Alterations in the tRNA pools due to modifications or changing cellular states lead to changes in translation elongation rate. Limitation of amino acids causes both global or codon-specific effects on translation levels and elongation speed. Nascent chain sequence features such as size and charge modulate translation elongation. Poly(A) tracks within the mRNA alter ribosome translocation rate triggering the RQC pathway and thereby reduce the net protein output. (*b*) Ribosome queueing/stalling occurs (i) when a ribosome encounters Pro-Pro codons in the mRNA sequence at the P:A-sites, (ii) due to slow release of ribosomes at the mRNA stop codon, (iii) due to the presence of certain combinations of amino acids like RxK at the E:P:A-sites or acidic amino acids like D and E at the P:A-sites. However, hypusinated eIF5A could rescue the ribosomes stalled at the Pro-Pro motif. (*c*) RQC pathway comes to the rescue when ribosomes stall due to defective mRNAs. EDF1 has been very recently identified to be associated with collided ribosomes which then leads to stabilization of GIGYF2-EIF4E2 complex on these stalled complexes thereby inhibiting further translation initiation of the defective mRNA. Upon persistent collision, ZNF598 ubiquitinates the 40S subunit triggering the RQC pathway where the nascent chain is degraded and the ribosome subunits are recycled.

A well-studied example of ribosome stalling happens during translation of poly-lysine tracks, in particular when ribosomes erroneously reach the poly(A) tail of the mRNA (figure 2*a*). Translation of the poly(A) tail can occur in defective mRNAs lacking a stop codon or in cases where a ribosome skips the stop codon. For a long time, it was unclear what triggered the stalling; the presence of lysine residues in the ribosomal exit tunnel caused by the decoding of the lysine AAA codon, or the presence of certain RNA-binding proteins in the poly(A) tail. A recent study [65] that solved the structure of the ribosome translating a poly(A) stretch, revealed that both the presence of multiple lysines interacting with the ribosomal exit tunnel and a consequent rearrangement of the conformation at the decoding centre play an additive role in promoting ribosome stalling in poly(A) motifs such as the poly(A) tail. Importantly, this work also showed the requirement for at least 10 consecutive lysine residues with at least the last two being coded by the AAA codon in order to promote stalling, showing selectivity of the ribosome for stalling in poly(A) tails.

Another trigger for ribosome stalling is amino acid limitation (figure 2*a*). While the limitation of some amino acids does cause ribosomes to stall during elongation, limitation of other amino acids can lead to a global shut down of translation initiation due to limited availability of aa-tRNA. This has been described in detail for arginine and leucine [66] where the loss of arginine tRNA charging resulted in ribosome pausing at two of the six arginine codons, reflecting not only an amino acid-specific but also a codon-specific stalling effect in elongation. On the other hand, the same work showed that a depletion in the levels of leucine is sensed by the mTORC1/GCN2 axis, leading to a shutdown of translation initiation. Therefore, this important work revealed alternative mechanisms used by cells to regulate translation in different nutrition-restricted states, favouring widespread translation arrest in the case of depletion of more essential amino acids such as leucine, while preferring a more specific regulation in cases of depletion of amino acids like arginine. It is interesting to note that the effects observed for arginine were codon-specific; this specificity was shown to reflect a regulation at the charging levels of different isoacceptor tRNAs, with the arginine tRNAs that decoded the pause-site codons showing a stronger loss of charging upon arginine limitation.

Moreover, in yeast cells, an additional trigger has been observed for specific codon pairs; it was found that 17 codon pairs strongly inhibit translation by slowing elongation, an effect that is also specific for codon order in each of these pairs [44]. Recently, a more detailed account of the inhibitory pair effect revealed that these codon pairs cause the ribosome to stall with an empty A-site which is caused by an alteration in the structure of the mRNA inside the decoding centre, creating an aberrant structure that precludes A-site tRNA accommodation [67]. It is so far not clear if this inhibitory pair effect is also present in mammalian cells; however, differences in elongation rate for certain codon pairs have been described in mammalian tissue [18].

A recent wealth of studies on ribosome collisions based on disome (or di-ribosome) sequencing has provided novel insights into these collision sites. Profiling of mRNA reads protected by two ribosomes, indicating collision events, has revealed the frequency and sequence context of these events. A study in yeast suggested that these collision (or ribosome queuing) events, which are due to translational pausing, are more frequent than previously thought, with one to five translating ribosomes involved in collisions [68]. In mouse liver, it was estimated that around 10% of ribosomes are in the disome state [69]. The frequency of these collisions was found to be related to the translational flux (protein synthesis rate). Moreover, the features of the amino acids being encoded, including charge and structure of the nascent polypeptide, were identified as causes of ribosomes pausing [69–71]. The slow release of ribosomes at the stop codons also led to ribosomal stalling [70,71] (figure 2*b*). A recent study focussed on proteins recruited to the collided ribosomal complex using a combination of polysome profiling and quantitative proteomics. EDF1 was identified as a novel protein, binding to collided ribosomes. It was also observed that EDF1 recruits and stabilizes the GIGYF2-EIF4E2 complex to the mRNA to inhibit further translation initiation of the defective mRNA [72,73] (figure 2*c*).

## 3. Clearing traffic jams and ribosome collisions

### 3.1. eIF5A: a codon-specific rescuer of ribosome stalling

In order to prevent unwanted ribosomal stalling, which could be detrimental for the translation efficiency of specific transcripts and for proteostasis in general, cells have found ingenious mechanisms. One example is the eukaryotic initiation factor 5A (eIF5A; (E)longation (F)actor-P in prokaryotes), which has been widely described to have an important function during translation elongation [63,74]. eIF5A, first extracted from rabbit reticulocyte lysate, was identified as a translation initiation factor using *in vitro* assay, as it facilitates the formation of methionyl-puromycin, representing the first peptide-bond formation (reviewed in [75]). However, later studies showed it has a substantial role in translation elongation. There are two isoforms of this protein found to be expressed in eukaryotes, eIF5A1 and eIF5A2, of which the former is more common and expressed in most tissues. There is also evidence suggesting altered regulation of these isoforms in various cancers [76,77].

eIF5A is the sole protein in the eukaryotic proteome to be identified to undergo the post-translational modification known as *hypusination* [78] at the lysine residue (Lys50 in humans and 51 in yeast). Both isoforms mentioned above are hypusinated. This is a two-step process coordinated by two enzymes called deoxyhypusine synthase (DHS) and deoxyhypusine hydroxylase (DOHH) [78]. Genetic and biochemical assays have shown that this hypusine modification is important for the function of eIF5A protein.

From earlier work in yeast and from functional homology studies with the prokaryotic EF-P, eIF5A was described to have a specific role in the elongation of poly-proline motifs [79]. When a poly-proline stretch is present in a protein, this could slow down elongation in that particular motif, creating a ribosome stall, where a ribosome with proline in both the P- and A-sites would have a free E-site. In this case, eIF5A is able to recognize the stalled conformation and enter the free E-site, in a way that the hypusine residue reaches the peptidyl transferase centre, stabilizing the prolines and facilitating the peptide-bond formation [80] (figure 2*b*). Recently, this function of eIF5A in elongation was confirmed in mammalian cells as well, where the depletion or ablation of hypusinated eIF5A levels resulted in increased specific ribosome stalling not only on poly-prolines but also in several other motifs that included codons for glycine, glutamate, aspartate, serine, lysine and leucine, among other [81].

Moreover, two recent independent works repurposed a role for eIF5A in translation initiation of upstream open reading frame (uORF) containing transcripts, in an elongation dependent mechanism [81,82]. In the models described, the coding sequences (CDS) of the uORFs contain eIF5A target motifs, so in the event that a scanning ribosome initiates translation of the uORF, the ribosome stalls in that motif and needs eIF5A to resume translation. If eIF5A is present, translation of the uORF is resumed and other scanning ribosomes are free to initiate translation in the main ORF. In the absence of eIF5A, the stalled ribosome translating the uORF will create a blockade preventing other scanning ribosomes to initiate translation of the main open reading frame (ORF), leading to a reduction of the expression of the respective protein. This mechanism was described in detail for only two transcripts, *azin1* and *myc*, but it is assumed that this could be a more widespread phenomenon [81,82].

These works also revealed the importance of eIF5A in regulating the maintenance of a particular proteome state through the regulation of translation elongation. When eIF5A is not active, specific sets of proteins, notably those related to extracellular matrix formation, cytoskeleton organization and cellular proliferation, are downregulated [63]. On the other hand, in cases when eIF5A is overexpressed, such as found in several cancers [83–86], these same signatures would have their translation efficiency increased, which could have implications for cancer progression.

### 3.2. Ribosome quality control/codon optimality dependent RNA degradation

We have described different signals that can cause stalling of a ribosome during translation elongation (tRNA landscapes, poly(A) tail translation, perturbed elongation cofactors such as eIF5A, truncated or defective mRNAs). If the stalling is not resolved in time, the trailing translating ribosome collides with the leading/stalled ribosome. This sort of collision can result in early release of truncated polypeptides, having detrimental effects for cells. A decade ago, a seminal work [87], started to shed light on a complex quality control pathway that is able to sense collided ribosomes, target the nascent proteins and the mRNA for degradation and recycle the ribosomal subunits, this pathway was called RQC. The importance of RQC is revealed by its conservation along evolution. Soon after an initial body of work which described in great details several steps of the RQC pathway in yeast cells, the components and mechanisms of this pathway started to be revealed also in mammalian cells [88–93].

The first step in RQC is the sensing of collided ribosomes and there are multiple sensing mechanisms to ensure efficient recognition of different types of defective translation. In the more classical case of translation of truncated mRNAs, the HBS1L/GTPBP2/PELO complex is able to sense translating ribosomes with neither mRNA nor charged tRNA in the A-site. PELO and HBS1L are paralogs of the eukaryotic termination factors eRF1 and eRF3, respectively, and are thus able to mimic translation termination of these faulty mRNAs. Upon PELO binding to the empty A-site, the ATPase ABCE1 is recruited, inducing the splitting of the two ribosomal subunits, the 40S subunit is recycled and the 60S subunit, still loaded with the tRNA-nascent chain complex is handed to the RQC complex for processing, in the next steps of the RQC pathway [94].

In the case of ribosome collisions occurring due to poly(A) sequences, it was shown that the ubiquitin ligase ZNF598 recognizes these collided ribosomes and ubiquitinates the 40S ribosomal proteins RPS10 and RPS20 [95,96], thereby targeting these ubiquitinated ribosomes for RQC. These actions seem to be coordinated with the ubiquitination of RPS2, RPS3 and RPS20 by RACK1 [97]. Recently, it was shown that the ZNF598-mediated ubiquitylation event can be reversed by USP21 and OTUD3, probably to avoid the degradation of the 40S subunit, allowing its recycling in further translation rounds [98]. The intermediate step between ZNF598-mediated ubiquitylation of the 40S subunit and the splitting of the two ribosomal subunits and following recruitment of the RQC complex is still not known.

In yeast, following the splitting of the two ribosomal subunits and recycling of the 40S subunit, the mRNA is targeted for degradation by Xrn1 and the exosome complex. However, a recent work [99] suggests that in mammalian cells this may not be the case, as they find little mRNA degradation in a ribosome collision reporter. Both in yeast and in mammals, the 60S subunit loaded with the nascent chain and the P-site tRNA are recognized by the RQC complex (figure 2*c*). The obstructed 60S subunit carrying the exposed nascent polypeptide along with peptidyl-tRNA is then specifically recognized and bound by NEMF, which in turn recruits LTN1, stabilizing its binding to the 60S subunit. LTN1 is an E3 ubiquitin ligase that polyubiquitinates the nascent polypeptide on lysine residues which is then targeted for degradation, while the now free 60S subunit can be recycled [94,100]. In some cases, where the nascent polypeptide does not have lysines accessible to LTN1, NEMF mediates a process known as CATylation (C-terminal alanine threonine tails), where alanine and threonine residues can be added to the nascent chain in a mRNA, thus extending the nascent chain and increasing the chances of a lysine residue to become accessible to ubiquitylation from LTN1 [93].

More detailed mechanisms and regulatory processes involved in RQC remain poorly understood. However, recent advances in ribosome profiling techniques, such as methods to study collided ribosomes (notably disomes, see above), will certainly provide more comprehensive insights into what triggers ribosome collisions and how the RQC is activated.

## 4. Mathematical models of translation

The described molecular complexity of the translation process makes the quantitative interpretation of translation experiments, in particular ribo-seq, highly challenging [56,101–103]. Before we introduce the mathematical models that are used to interpret ribo-seq data, we briefly review some specifics of the experimental protocols that matter for analysis.

The first step of ribo-seq involves halting translation using translation inhibitors or flash-freezing; then, the mRNA together with the bound ribosomes is isolated and undergoes nuclease digestion. Fragments of about 30 nucleotides (corresponding to the typical length of ribosome protected fragments, called ribosome footprints) are selected for DNA library preparation and sequencing. Finally, reads are mapped to the genome and the A-site of the ribosome is identified for each read, yielding the position of the translating ribosome at nucleotide resolution. The reads corresponding at each position are finally summed, yielding the ribosome occupancy profile for each gene [104].

In practice, the experimental protocols are prone to biases which represent an important confounding factor in the analysis. For instance, the use of cycloheximide to arrest translation immediately prior to RNA extraction can significantly affect the coverage profile, notably by causing accumulation of ribosome density near translation start sites and ‘smearing’ of ribosome density in gene bodies [105,106]. Indeed, there is evidence that translation continues in presence of cycloheximide but codon-specific elongation rates are dramatically altered [107], while in mouse liver, the use of CHX in the lysis buffer does not change the translation dynamics [18]. Library preparation can distort the experimental result as well, by including a number of reactions involving enzymes with nucleotide sequence specificity, such as digestion and ligation [101]. In some datasets, these sequencing biases have a greater influence on the coverage pattern than the identity of the codons in the decoding centre of the ribosome [37]. The current ribo-seq workflow usually selects reads of a specific length (approx. 30 nt). This has the advantage of reducing ribosomal RNA contamination, but it can hide part of the translation response. In particular, pairs of stalled ribosomes may protect longer mRNA fragments from nuclease digestion, so that data may be depleted of queued ribosomes [37]. The data analysis can introduce additional uncertainty, in particular in the identification of the position of the A-site in the ribosome footprint. This is indeed a crucial step in the analysis and a number of methods have been developed [38,42,64,107–112] to provide a reliable estimate of the position of the ribosome at codon resolution.

In recent years, mathematical and computational models of translation have become a crucial ingredient for interpreting ribo-seq data. Such a complex and multi-sided problem has attracted researchers from different domains and motivated the development of specific techniques to investigate the determinants of translation elongation and protein synthesis rate.

### 4.1. Totally asymmetric exclusion process models

**Figure 3.**
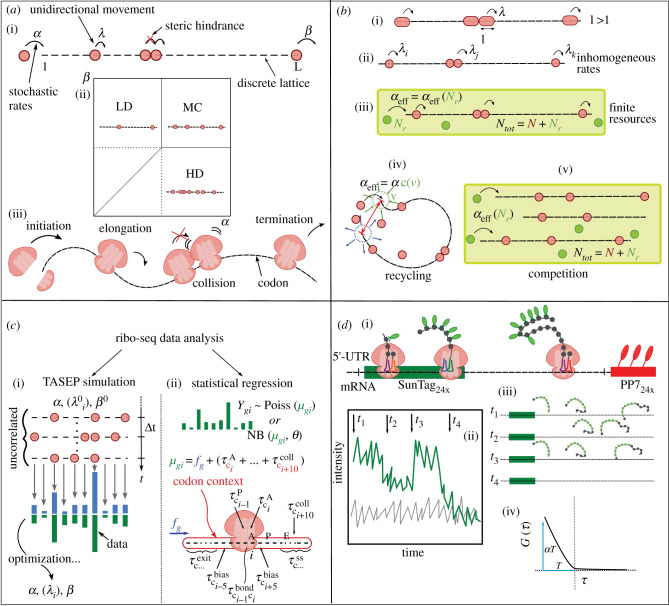
Theoretical modelling and analysis of translation. (*a*) (i, ii) schematic representation of the totally asymmetric exclusion process (TASEP) with particles of size ℓ = 1 and its phase diagram; the three steady state regimes are shown (low density (LD), high density (HD) and maximal current (MC)). Initiation, termination and elongation rates are indicated respectively by *α*, *β* and *λ*. (iii) simplified representation of the translation of an mRNA molecule, pointing out links to the TASEP. (*b*) Generalizations of the TASEP: (i) extended particles (ℓ > 1); (ii) inhomogeneous rates *λ_i_* ≠ *λ_j_*; (iii) finite resources, corresponding to the TASEP with a total number of particles *N_tot_* which is fixed, shared between the reservoir (*N_r_*) and the lattice (*N*); (iv) recycling mechanisms: particles can diffuse in a three-dimensional reservoir, the lattice is modelled as a flexible polymer; the effective initiation rate *α_eff_* depends on the concentration of particles in a local volume *v* around the initiation site; (v) competition for resources: multiple lattices (possibly of different lengths) compete for a finite number (*N_tot_*) of particles. The effective initiation rate *α_eff_* depends on the number of particles available for initiation (*N*). (*c*) Analysis of ribo-seq data by means of TASEP simulations (i) and generalized linear regression (ii). (i) Simulation results and data are compared and the input parameters are modified to match the data. (ii) Under a low-density assumption, the logarithm of the mean density (*μ_gi_*) of reads at each position is approximated by the sum of the logarithm of the gene-specific flux (*f_g_*) and the logarithm of the dwell time; the latter is the sum of different parameters, accounting for decoding time at A-site, peptide-bond formation, exit-tunnel interactions, collisions and bias. A generalized linear model is fitted to the data. (*d*) (i) Schematic of the SunTag reporter mRNA. Repeat-epitope tag called SunTag (dark green mRNA) encodes for peptides (grey hexagons) which can be co-translationally labelled by fluorescent antibodies (green ovals). An array of MS2 stem-loops (red mRNA) in the 3′ UTR allows visualizing single mRNAs with a fluorescently tagged MS2 coat protein. (ii, iii, iv) In the larger plot, ideal fluorescent light intensity traces of a transcribed reporter mRNA in time; different time points (*t_1_*, *t_2_*, *t_3_*, *t_4_*) correspond to the configurations of translating ribosomes reported on the right. The smaller plot is a schematic representation of the profile of the auto-covariance function *G*(*τ*) of the light intensity signal as a function of time delay *τ*. The time at which *G*(*τ*) goes to zero estimates the characteristic time *T* for a ribosome to translate the gene from the tag region to the end of the protein of interest. From *G*(*τ*) both the initiation rate and the total dwell time can be estimated. The method is known as fluorescence correlation spectroscopy (FCS) and it is one of the methods used to infer translation time together with run-off assay (ROA) and fluorescence recovery after photobleaching (FRAP).

A significant step was to reduce the complexity of translation to a dynamical model with a small number of parameters. One successful model, which captures a number of essential properties of translation (discrete and uni-directional steps, steric hindrance, stochasticity), is known as the TASEP, which belongs to a wider class of processes studied in statistical physics called interacting particle systems. In biology, it was introduced by Gibbs & co-workers [113], to describe the dynamics of ribosomes translating an mRNA transcript; contemporarily, it was introduced in mathematics by Spitzer [114] to study processes of Brownian motion with hard-core interactions. In essence, the TASEP consists of particles moving along a one-dimensional lattice by hopping from one site to the next with a constant rate [115]. A-site can be occupied by only one particle at a time; therefore, a particle can hop forward only if the next site is empty (figure 3*a*). Over the years, the model revealed itself to be suitable to describe a variety of phenomena, from road traffic [116] to biological transport [117,118].

In the original TASEP, particles have unitary length (they cover only one site) and hopping rates are equal for each site of the lattice (homogeneous model) (figure 3*a*). Boundaries may be closed (periodic boundary conditions) so that first and last sites coincide, or open. The latter case is indeed more biologically relevant for translation. With this choice, each boundary is connected to an infinite reservoir of particles; particles (e.g. ribosomes) enter the system at the first site, go through the entire lattice and exit the system at the last site. In a biological context, the entry rate is usually referred to as the initiation rate, the exit rate as the termination rate and the hopping rates as the elongation rates. In this original formulation, elongation rates are all equal, while initiation and termination rates may be different (all of them are constant in time). Depending on the value of these two parameters, the system can be found in three steady state phases: a half-filled phase called maximal current (MC), a low-density phase (LD) and a high-density phase (HD) [115] (figure 3*a*). Each phase is characterized by different relationships between the current and the density of particles.

More complex formulations of the original TASEP have been extensively studied in recent years as more realistic models for protein synthesis (see [115,117] for reviews on these models and their applications). The steady states of such models are typically not known analytically and they are obtained via approximations or Monte Carlo simulations. Such models have been extensively used in the interpretation and analysis of ribo-seq data, especially to infer initiation and elongation rates [7,11,56,119–123] and to quantify ribosome queueing [68]. In addition, the study of these theoretical models has shed light on the main features of the codon sequence determining protein synthesis rate [10]. They have been applied to ribo-seq data mainly in yeast (*S. Cerevisiae*), more rarely in mammals [56,123].

### 4.2. Generalizations of the totally asymmetric exclusion process

A first, important, generalization of the TASEP was made by Gibbs & co-workers [113,124] who considered particles of size greater than 1 (ℓ > 1) (figure 3*b*(i)), to take into account the length of a ribosome, which covers approximately 30 nucleotides (10 codons) [64,125]. While in the original formulation (ℓ = 1, homogeneous elongation rates) of the model, currents and densities can be computed exactly for any value of the initiation and termination rate [126–128], the extension to particles of arbitrary size (ℓ > 1) has no exact solution. Some good approximations exist, inspired by mean-field approaches [113,129] and perturbative approaches [130]. The phase diagram of the TASEP with ℓ > 1, characterized via Monte Carlo approaches and mean-field approaches [131], is qualitatively similar to the one of the original TASEP, in particular it shows the three phases (figure 3*a*).

The simplest approximation assumes that the density of ribosomes is sufficiently low that ribosome collisions can be neglected, significantly reducing the complexity of the model. The approximation is often used in the interpretation of ribo-seq data, since there is evidence that translation mostly happens in a low-density regime, for example in wild-type yeast [10], though it is still debated how important collisions are in different systems under different conditions [69,71,132]. When it is valid, the density of reads at each codon can be approximated by the flux (gene specific, equivalent to the protein synthesis rate per mRNA) divided by the elongation rate (specific to the codon being translated and to the codon context) (for a detailed explanation, see the supplementary material of [119]). Using this approximation, and assuming that ribosome drop-off is negligible during translation (so that the flux is conserved along the transcript), it is possible to estimate the dwell times (reciprocal of elongation rates) directly from the number of ribo-seq reads, up to a gene-specific proportionality factor. Indeed, under this assumption, the dwell time at each position along the CDS is proportional to the number of ribosome protected fragments at that position. In practice, one also needs to account for experimental bias which may confound the dwell times [18].

A second approach is to use mean-field (MF) approximations. Mean-field approaches involve neglecting certain correlations in the system by assuming that the full probability distribution of the model factorizes into a product of local marginals [133]. The ordinary MF assumes that the marginal probability distribution for site *i* and *i* *+* ℓ being occupied factorizes in the product between the marginal probability for *i* being occupied *and* the marginal probability for *i* *+* ℓ being occupied. Here, ℓ represents the size of a ribosome, such that a ribosome at *i* can move one codon forward only if there is no ribosome at position *i* *+* ℓ (occupying the ℓ codons downstream).

Unfortunately, for TASEP with ℓ > 1 the simple MF approximation is very poor [117]. A more sophisticated approximation (also ‘mean field', since it neglects some correlations) has been developed [124,134], predicting more accurately the average current. An original approach [129] takes advantage of the fact that, in the ‘bulk' (far away from the boundaries), the system in MC and steady state can be described as a gas of particles in a one-dimensional discrete lattice with hard-core interactions. Using the statistical mechanics theory for such gas in the MC phase and a refined mean-field approach for the LD/HD phases, they found values of the current in agreement with Monte Carlo simulations (within numerical precision). Svazits-Nossan *et al.* [130,135] developed an initiation-limited approximation for the densities and the currents based on the expansion of the model probability distribution in powers of the initiation rate. This approximation is based on the assumption that initiation is the rate-limiting step of translation. They found the expansion coefficients up to third order, exploiting a graph-theoretical interpretation of Markov chains applied to the TASEP.

A second important extension of TASEP is to consider inhomogeneous elongation rates. As already mentioned, elongation rate heterogeneity is due to multiple factors, such as tRNA abundance, peptide-bond formation, mRNA secondary structure, exit-tunnel interactions [119] and co-translational folding [136]. Heterogeneity of elongation rates is indeed suggested by ribo-seq profiles, representing (as discussed before) estimates of the dwell times [119] under low-density assumptions.

The McDonald and Gibbs mean-field approach, applied by the authors to the uniform system, is effective also in describing non-uniform systems [137]. When the latter approach fails, extending the original mean-field approach to include two-point marginals (and factorize higher-order marginals) is more effective in describing the system than the ordinary, one-point mean field, but it is also more prone to numerical instabilities and analytical difficulties [137]. In general, all the approximations described for the uniform case have been generalized to the TASEP with non-uniform rates, except for [129]. This approximation relies on the equivalence of the system in the MC phase with a gas of particles at equilibrium, based on the fact that in an infinite lattice at maximal current the particles are uniformly distributed in the bulk. This is no longer true when the rates differ from site to site. Also their MF approximation for the boundary-limited regimes is based on the assumption of nearly uniform distribution of particles and may not be suitable to be generalized to an arbitrary set of rates.

Recently, a solution for the TASEP model with extended particles and inhomogeneous rates was developed [10], which is valid in the *continuum* limit (hydrodynamic limit), when the rates can be described by a ‘smooth’ function of position along the transcript. Even if real rates are highly discontinuous from codon to codon, their approach has shed light to the main features of codon sequence determining translational efficiency, by unravelling the density-current relations in different regimes of translation. They have found the existence of the same steady state phases of the simple TASEP (LD, HD and MC) and quantified the importance of sequence-dependent features such as the position of the minimum rate and the elongation rates near the beginning of the ORF. In particular, if the minimum rate is located in the 5′ region of the ORF it allows the number of ribosomes to be reduced and hence the cost of translation in the MC phase. The elongation rates near the beginning of the ORF also play an important role, since high elongation rates increase the sensitivity to the initiation rate, a useful property for highly expressed genes or regulated genes which are subject to variable demand in expression levels.

From the analysis of the elongation rates inferred in a preceding work [119], the authors [10] found that these principles are in general observed in yeast. For example, the 5′ translational ramp, which represents a pattern of translational slow-down around codon position 30–50, may serve to prevent crowding of elongating ribosomes by placing the minimum rate early in the sequence, as precedently suggested [138]. In addition, the theoretical analysis helps answer the long-lasting debate about codon usage bias, which is the enrichment of specific synonymous codons in highly expressed genes. Indeed, this has been observed in yeast, suggesting the hypothesis that codon usage bias accelerates elongation [139]. According to [109], this mechanism should have a significant impact on translational efficiency only when affecting the location of the minimum rate and the rates in the early ORF. In all the other cases, however, it could impact protein production rate indirectly by reducing ribosome density on the transcript and, hence, the cost of translation. This would in turn increase the availability of free ribosomes, ready to be recycled for a new turn of translation. We will discuss the aspect of recycling later in the section.

Inferring biological elongation rates from experimental measurements to assess translational efficiency remains highly challenging. A few works [18,38] inferred codon-specific dwell times and gene-specific fluxes by means of statistical regression and by assuming that ribosome density is sufficiently low to neglect collisions. Based on this assumption, they consider the density of reads at each position as a product of flux and dwell time, where the dwell time can be a product of sequence-specific parameters, which depend on the codon context and that may include features such as exit-tunnel interactions, mRNA secondary structures, the identity of the codon being translated as well as technical bias. In order to fit the parameters generalized linear models are used, with a suitable noise function (such as negative binomial distributions [18]) or likelihood penalty to account for sampling bias [38] (figure 3*c*).

Under perturbation, notably the depletion of a specific amino acid (3-AT conditions), one of these models [18] captured signatures of collided ribosomes, by including additional ‘collisions' parameters revealing ‘shadows' in the dwell times patterns offset by one ribosome. This suggests that, in certain conditions, ribo-seq analysis of monosomes can detect ribosomes collisions, even though the mRNA cleavage between adjacent ribosomes is likely less efficient in the presence of two (or more) stacked ribosomes and therefore the signature of collision may be underestimated in monosome data.

These methods based on statistical regression do not include interactions between particles (contrarily to TASEP), hence collisions can be detected only partially and where they appear systematically (like in 3-AT conditions). Nevertheless, these methods are likely to predict dwell times reliably in wild-type conditions, for which low density is in general regarded as a realistic assumption. The advantages of the low-density limit with respect to more accurate schemes relying on simulations presented previously are the smaller cost in terms of time and computational resources and, importantly, the possibility to fairly easily account for experimental bias when inferring dwell times. This last feature is particularly relevant as ribo-seq can be subject to experimental protocol-specific bias (in particular, during cleavage and library preparation) which needs to be carefully taken into account during the analysis, and which is often disregarded when analysing the data using TASEP-based theoretical approaches [18,37,101].

Recently, some studies have revealed the presence of ribosome collisions in yeast [68,70,140] as well as in mammals [69,71], suggesting that dynamical models such as TASEP are likely to be more suitable to inquire translation and its regulatory mechanisms. In the presence of collisions, the relation between dwell times and ribosome density is nonlinear and complex; to deal with this complexity several authors used the TASEP to simulate translation [119,120,122]. The simulation is usually based on a Gillespie algorithm where the elapsed time for each event is drawn from an exponential distribution with mean the inverse of the rate. Simulating the process requires fixing a large number of parameters, namely the elongation rates and the initiation/termination rates. The simulation is used to verify the agreement of the predicted rates and the experimental data; it is also used to optimize the parameters to match the data (figure 3*c*). In [119], for example, the authors infer the rates from the density profile by optimizing the match between the simulated profile and the experimental profile; other authors [120] infer the rates by optimizing an objective function based on the initiation-limited approximation discussed before, or use an iterative optimization procedure to obtain the best agreement between the simulated profile and the data [122]. The inferred rates may be transcript- and position-specific [119,120], or codon-specific (independent of the specific transcript) [122]. An important drawback of these approaches is that they are computationally expensive and time-consuming, so that some authors have developed a coarse-grained version of the TASEP model, requiring fewer computational resources and allowing for a simpler analytical treatment [121].

An important biological feature of translation, not included in the models presented above, is that the pool of resources (such as ribosomes and tRNAs) each mRNA have access to is *finite*. A variant of the homogeneous ℓ = 1 TASEP [141] introduces a global constraint on the number of particles (ribosomes) (figure 3*b*(iii)). Namely, the boundaries and the system share a fixed number of particles, so that an increase in the number of particles in the system determines the depletion of the reservoir. In the model, the constraint on the total number of particles acts indirectly on the initiation rate, which depends on the number of particles in the reservoir and decreases as the reservoir is depleted. Constraints on the number of ribosomes may be relevant when considering rapid cell growth, when resources may become rate-limiting; it would be interesting to consider the effect of constraints on the number of aa-tRNAs, which may be relevant in nutrient-limiting conditions.

Above, we have briefly mentioned recycling as a possible feedback mechanism enhancing translation. Recycling mechanisms may indeed have an important role in regulating the translation of highly expressed genes. In fact, it is thought that some components of the translational machinery can be recycled after termination, without completely re-entering the enzyme pool in the cytoplasm, thus increasing translation efficiency [142]. To study this phenomenon, some works [143,144] have coupled the TASEP with ribosome diffusion in a three-dimensional medium, and these model ribosome adsorption/desorption kinetics at mRNA initiation/termination sites as well as polymer physics, which determines the spatial distribution of the termini with respect to each other (figure 3*b*(iv)). This process can be facilitated by additional mRNA-binding factors which can prompt loop formation, bringing the 3′ and 5′ ends of the transcript in proximity [145–147]. This effect is modelled by binding energy between the 5′-cap and poly(A) tail proteins, determining the probability that the chain is looped and enhancing the ribosomal binding rate at the initiation site in the presence of cooperative effects [143].

All the TASEP-based models presented up to now consider one mRNA molecule at a time, completely neglecting the interactions among different mRNA molecules arising from competition for a finite pool of resources, such as ribosomes, translation factors and aa-tRNAs. First attempts to shed light on the competition for ribosomes have been made for homogeneous TASEPs with particles of unitary size (ℓ = 1). The authors have solved the steady state of a system with an arbitrary number of TASEPs connected to a common finite particle reservoir [148] (figure 3*b*(v)). A further generalization is needed for the model to be relevant for protein synthesis, among which those already mentioned (particles of a size larger than one lattice site, inhomogeneous elongation rates, recycling) and competition for aa-tRNAs and binding factors.

### 4.3. Single-molecule analysis of translation

Ribo-seq provides information about the translation process averaged over thousands of mRNA molecules and cells and, despite being a powerful tool, it is not suitable to measure certain aspects of translation heterogeneity [149]. For example, each transcript of a gene can undergo translation with different starting sites or even different frames, giving rise to canonical and non-canonical translation [150,151] (intra-genic heterogeneity). In addition, translation inside the cell can be regulated differently in time and space, as in primary neurons, where mRNAs are translated in proximal dendrites but repressed in distal dendrites and display ‘bursting' translation [6]. Finally, if differences are present among cells in a tissue (inter-cellular heterogeneity) they would also be averaged out in a ribo-seq experiment.

These sources of heterogeneity have been neglected in most TASEP-based theoretical models, also because their authors have global measurements, specifically ribo-seq and rna-seq, as experimental reference. Even the regression approach makes strong assumptions of homogeneity: in particular, the fact that each transcript of a gene is translated in the same conditions (with same initiation rate and protein production rate), and that dwell times are equal across different transcripts.

Measurement of translation at single-molecule level has become possible thanks to recent advances in protein tagging and imaging techniques. Among the recently developed assays, TRICK allows the distinction between mRNAs having already undergone a round of translation and the untranslated ones [152]. Another set of assays, single-molecule imaging of nascent peptides (SINAPS), allow monitoring of translation of single mRNA molecules *in vivo*. In these assays, a reporter mRNA is modified to encode multiple epitopes in the open reading frame of a protein of interest. When the protein is translated, the epitopes are recognized and bound by fluorescent antibody fragment probes (figure 3*d*). The use of this construct in combination with MS2 tagging—used for image detection of mRNA in living cells—allows single-mRNA molecules undergoing active translation to be monitored [153]. It is possible to track translation dynamics over time and use the data to quantify the total time needed to translate the open reading frame as well as the translation initiation rate (see [154] for the fluorescent signal analysis and [155] for review). By combining orthogonal fluorescence labelling systems, such as SunTag [156] and MoonTag [150], it is possible to track start site selection and selection of different reading frames, largely expanding the possibility to investigate translation heterogeneity at the single-cell level.

In a recent study [153] the TASEP was further generalized to include the arbitrary placement of fluorescent probe-binding epitopes, in order to mimic the result of imaging single-molecule translation dynamics. By using the stochastic model to simulate realistic synthetic data, the authors could compare different experimental assays and establish which, among them, is more likely to provide reliable estimates of kinetics parameters. They calibrated their stochastic model using fluorescence correlation microscopy (FCS) data (the most reliable assay according to their analysis) to estimate initiation and elongation rates (figure 3*d*(ii), (iii), (iv)). The authors provided an open-source software package (rSNAPsim) to simulate the output of different types of single-molecule experiments (see caption of figure 3*d*), for any gene sequence and with different assumptions regarding synonymous codon usage, tRNA level modifications or ribosome pauses. Signatures of ribosome queueing have been recently observed in HIV-1 frameshifting translation sites by means of single-RNA imaging technique based on the combination of two different types of epitopes (SunTag and HA epitopes) and ribosome run-off assays [157].

So far, TASEP-based modelling of translation has been often applied to the analysis of genome-wide data, notably ribo-seq, revealing itself to be a powerful theoretical tool. The original model has been improved to include biological relevant features, such as ribosome size, heterogeneous elongation rates, finite resources and recycling mechanisms. The effect of finite resources, recycling and competition among mRNA molecules has been only partially explored, remaining an interesting future subject for research. A recent study [158] attempted to study translation at the whole-cell level, by simulating translation of thousands of mRNA molecules, including competition for ribosomes, tRNAs, tRNAs wobble interactions and tRNA recycling in *E. coli*, and represents a step towards studies of translation inclusive of multiple features at cell level. Combining different experimental assays in a synergistic way, such as single-cell and single-molecule measurements as well as global measurements, may provide unprecedented opportunities to understand translation in depth.

Single-molecule simulations of translation can aid the interpretation of experimental data; in particular, the combination of TASEP-based modelling and single-molecule time-resolved assays could shed light on the dynamics of translation as well as its heterogeneity and competition for resources at the cellular level. TASEP modelling could be further complexified in order to take into account features of interest, such as three-dimensional structure of polysomes (which could influence translation, also through particle recycling) which can be visualized thanks to electron-microscopy techniques [159], non-canonical translation, frameshifting, all measurable using SunTag/MoonTag systems, for example. In addition, theoretical modelling could benefit from direct measures of translation initiation and total dwell time provided by single-molecule imaging.

## 5. Conclusion

Production of functional proteins is a prerequisite to the proper functioning of various cellular processes. A growing body of evidence suggests that translation elongation is a critical process modulating translational yield of an mRNA by various feedback mechanisms. In this review, we highlight some of the key molecular determinants of translation elongation in mammals, unravelled by recent studies. Although methods like ribosome profiling and tRNA profiling along with theoretical modelling have revealed various factors affecting the dynamics of translation elongation, there is still a lot that needs to be uncovered. For example, ribosome dwell times are highly variable from codon to codon and specific codon pairs are known to slow-down translation elongation. Reasons behind these are still largely unknown. However, a combination of above-mentioned approaches along with structural and single-molecule studies would shed light on this.

On the theoretical side, TASEP-based approaches are powerful tools to interpret ribosome profiling data, since they can fully account for the dynamics of the process. However, they require complex simulations and the tuning of many parameters. Statistical analysis-based approaches, such as the generalized linear model, provide insights on the determinants of the elongation rates and require much less computational resources, with the important drawback of only partially accounting for collisions and of assuming the existence of genome-wide dwell times.

Translation in mammals is a complex process, which is still less well-understood than in bacteria and yeast. An interesting perspective for the future would be to extend the study of mammalian translation by incorporating information coming from single-molecule measurements of translation. This would allow monitoring translation at the single-cell level and *in vivo*, opening the possibility of measuring directly the initiation rate as well as the total elongation time of each transcript. We envisage that the combination of different assays, in bulk as well as in single molecules, along with the mentioned theoretical approaches, will provide new possibilities of understanding the translational process in depth, as well as its perturbations during stress or disease.

## Data accessibility

This article has no additional data.
